# Fabrication of Collagen–Hyaluronic Acid Cryogels by Directional Freezing Mimicking Cartilage Arcade-like Structure

**DOI:** 10.3390/biom12121809

**Published:** 2022-12-03

**Authors:** Taiyo Yamamoto, Rotsiniaina Randriantsilefisoa, Christoph Martin Sprecher, Matteo D’Este

**Affiliations:** AO Research Institute Davos, Clavadelerstrasse 8, 7270 Davos, Switzerland

**Keywords:** cryogel, cartilage, collagen, hyaluronic acid, double network, directional freezing

## Abstract

The internal architecture of tissue-like constructs is fundamental to their structural and biological functions. Here, we introduce a simple and robust method to fabricate cryogels based on derivatized extracellular matrix (ECM) macromolecules with porosity arranged according to the typical Benninghoff zonal architecture of articular cartilage. To obtain this arcade-like structure, the technique used the growth of ice crystals from copper pins at cryogenic temperatures. The directional cryogel formation enabled the organized growth of ice crystals over a large distance (>4 mm). The compositional properties were achieved by forming double networks (DNs) of hyaluronic acid and collagen derivatives (MeHA and CollGTA, respectively), which also served to improve the mechanical properties of the otherwise weak collagen scaffolds. Compositionally biomimetic and more resilient MeHA-CollGTA DNs (Young’s modulus ≈ 200 kilopascals) were therefore produced. The technique presented expands the fabrication methods available for providing ECM macromolecules with architectural elements mimicking cartilage complexity.

## 1. Introduction

Architecture and internal organization of biological tissues is tightly correlated to their properties and functions. One prominent example of this concept is articular cartilage, in which collagen fibers are organized in a typical arcade-like fashion, first described by Benninghoff [1]. In this structure, three zones can be identified: the superficial zone with densely packed fibers aligned parallel to the articular surface, the middle zone with obliquely arranged fibers, and the deep zone with fibers perpendicular to the surface [2,3]. This architecture is not only integral to the mechanical properties and functioning of articular cartilage, but also provides important depth-dependent topographic cues to chondrocytes, which adapt their morphology and expression patterns in response [4,5].

Different approaches have been used in attempts to recreate the zonal structure of articular cartilage in tissue engineering, each with specific strengths and limitations. Electrospinning is a widely used method to create scaffolds with aligned fibers. For instance, aligned fibers of electrospun polycaprolactone allowed the orientation and elongation of mesenchymal stromal cells (MSCs) [6]. Moreover, the use of aligned nanofibers significantly enhanced glycosaminoglycan (GAG) deposition in the presence of differentiation cues compared to microfibers or randomly oriented scaffolds [6]. Recently, Schwab and Hélary et al. introduced a 3D printing strategy using a hyaluronan bioink containing fibrillated collagen. The shear forces during the extrusion-based printing process were exploited to control the collagen fibers’ orientation on a microscopic scale. Consequently, MSCs spread according to the printed fibers’ direction [7]. Another technique is the growth and self-assembly of MSCs and chondrocyte spheroids through hydrophobic microchambers, leading to the creation of stratified cartilage constructs. The technique consists of forming a polycaprolactone lattice first in which the wells are filled with a gelatin methacrylamide (GelMA)-spheroid bioink. The spheroids rapidly condense and the lattice grows and eventually fuses into structurally organized cartilage tissues [8].

While these approaches show promising results, this intrinsic complexity leads to slow manufacturing, low throughput, limited scalability, and high costs. Therefore, mimicking the arcade-like structure of articular cartilage would benefit from simple and robust manufacturing processes.

Cryogelation is a highly tunable process where pore size and interconnectivity can be precisely controlled through factors such as freezing temperature, pH, or buffer composition [9]. Additionally, this versatile method can be applied to a wide variety of polymer–crosslinker combinations [10]. In this process, the solvent forms porogenic ice crystals and the polymer–crosslinker solution becomes locally concentrated, leading to crosslinking and cryogel formation [11].

**Figure 1:**
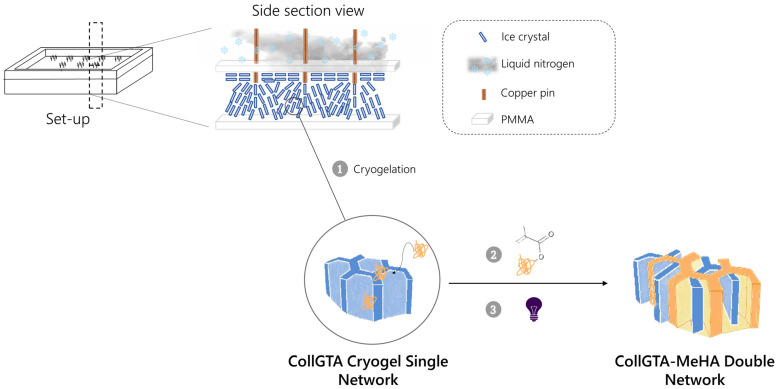
Schematic representation of the pin set-up for directional cryogel formation of a CollGTA first network by cryogelation (1) and the subsequent double network formation with diffusion of MeHA (2) and crosslinking by UV (3). The set-up is fully insulated and the copper pins in contact with liquid nitrogen allow ice crystals to grow from the pins, resulting in arcade-like structured cryogels.

The method introduced here is based on a directional freezing process combined with subsequent cryogelation (Figure 1). During the directional freezing step, an arcade-like structure is introduced into a scaffold through freezing using a newly developed pin plate set-up. Combining the directional pin freezing method with cryogelation locks this structure in place and therefore leads to a versatile and easily scalable method for mimicking the arcade-like structure of articular cartilage.

In this work, we hypothesized that simple copper pins in contact with a cold source could directionally guide the formation of ice crystals, mimicking in this way the typical geometrical arrangement of articular cartilage fibers. Furthermore, the porous structure lends itself to diffusion of additional components for generating composite structures with controlled architecture. This concept was tested by forming a DN based on semi-synthetic derivatives of the articular cartilage components hyaluronic acid (HA) and collagen.

## 2. Materials and Methods

### 2.1. Materials

HA with an MW of 63 kDa was purchased from Contipro Biotech S.R.O. Collagen type I from rat tail at a concentration of 10.57 mg/mL was purchased from Corning^®^ (Corning, NY, USA). All chemicals were used without purification unless otherwise noted.

The ^1^H NMR analysis was performed at 500 MHz on a Bruker Avance™ AV-500 NMR spectrometer. The chemical shifts were reported in δ (ppm) values and referenced to the indicated deuterated solvents. The samples were measured at a concentration of 15 mg/mL in D_2_O.

### 2.2. Methacrylate Hyaluronic Acid (MeHA) Synthesis

HA was functionalized with methacryloyl groups as described by Loebel et al. [12]. Briefly, 10 g of HA was dissolved in deionized water by stirring vigorously with a magnetic stir bar at RT. The dissolved HA was placed into an ice bath in a round-bottom flask. A pH-meter probe was placed into the HA solution and the pH was adjusted to 8.5 with 1 M NaOH. While stirring, 2.8 mL of methacrylic anhydride per gram of HA was added dropwise while regularly readjusting the pH to 8.5. Still in the ice bath, the reaction was kept at a pH of 8.5 through regular re-addition of 1 M NaOH for 4 h. The reaction was then left to complete overnight at RT while vigorously stirring. To remove residual methacrylic anhydride and formed methacrylic acid, the solution was dialyzed against deionized water at RT for 7 days, changing the water at least twice a day. The solution was then frozen, lyophilized, and stored at −20 °C until its use.

The degree of substitution of HA with methacryloyl groups was determined through ^1^H-NMR spectrum analysis (Supplementary Materials Figure S2). First, 2.5% (*w*/*v*) MeHA was dissolved in deuterium oxide containing 0.04% (*w*/*v*) hyaluronidase. Hyaluronidase digestion reduced the viscosity of the sample and was carried out at 37 °C for 24 h. The degree of substitution was calculated by normalizing with the integrals of the three methyl protons of the N-acetyl group (at ~2 ppm) and determining the relative ratio of the two acryl protons of the methacryloyl groups (two peaks between 5.5 and 6.5 ppm). The average degree of substitution over the two acryl peaks was 45%.

### 2.3. Pin Plate Set-Up

Schematics and build plans of the pin plate set-up are shown in Supplementary Materials (Figure S1). The pin plate was produced to fit on top of a custom Teflon mold plate with 13 cylindrical wells of a diameter of 10 mm and a depth of 6 mm. The bulk of the pin plate was built from glued 5 mm thick PMMA plates, creating a hollow structure that fits onto the Teflon mold plate on the bottom side and acts as a recipient for liquid nitrogen on the top side. Then, 0.4 mm wide holes were drilled across the plate and copper wires of the same diameter were fitted into the holes, as shown in Figure S1. The hollow structure was then filled with expanding insulation foam (Sika).

### 2.4. CollGTA First Network Directional Freezing and Cryogelation

First networks of CollGTA were prepared as follows. On ice, the pH of concentrated collagen was risen to 7 by addition of NaOH. The solution was then diluted to the required concentration by addition of PBS. A concentration of 0.1% (*v*/*v*) of glutaraldehyde was subsequently added to the mixture. The polymer–crosslinker solution was then pipetted into the Teflon molds, and the samples were frozen with liquid nitrogen in the pin plate set-up for 10 min. After that, the whole set-up containing the frozen first network solution was placed at −20 °C for complete cryogelation, taking place overnight. The obtained samples were all 1 cm in diameter and 6 mm thick.

### 2.5. CollGTA-MeHA Double Network Formation

CollGTA-MeHA double networks were formed by diffusion of MeHA second network solution into the CollGTA first networks and curing under UV light as follows. The CollGTA cryogels were immersed in a constant volume of fixed concentrations of MeHA solutions, typically 2, 3, 4 and 6% (*w*/*v*), and in the presence of Irgacure 2959 (0.5% (*w*/*v*) in PBS). This solution was placed on a shaking plate at 37 °C for 48 h. After that, the suspension and the cryogels were cured under UV light (80 J/cm^2^) for 15 min, forming CollGTA-MeHA DNs. The obtained CollGTA-MeHA DNs were subsequently washed in PBS until reaching equilibrium.

### 2.6. Swelling Testing

For swelling tests, cryogels (500 μL) were prepared as follows. First, 2 mL of PBS was applied to the cryogels, which were then incubated for several hours until reaching the swelling equilibrium. The surface water was then removed from the samples by using a blotting paper and the cryogels were weighed (ms). Then, the swollen cryogels were freeze-dried and weighed again to obtain the dried mass (md). The experiments were performed in triplicate and the mass swelling (q) was calculated with Equation (1):

q = (ms − md)/md(1)

### 2.7. Scaffold Morphology

#### 2.7.1. Light Microscopy and OrientationJ Analysis

Thin cross-sections of the scaffolds were cut out with a sharp blade and placed onto a glass coverslip. Light microscopy imaging was performed on a microscope (Axiotech, Zeiss, Jena, Germany, 5× objective, Axiocam 105). The samples were immersed in PBS during measurement to prevent drying and bubble forming. The Microsoft Image Composite Editor AxioVision SE64 Rel.4.9.1 (Axiotech, Zeiss, Jena, Germany) was used to stitch the individual images into composite images of the entire scaffold cross-section.

The OrientationJ plug-in for ImageJ developed by the Biomedical Imaging Group at the École Polytechnique Fédérale de Lausanne (EPFL) was utilized to analyze the angular orientation of the polymer fibers.

#### 2.7.2. Scanning Electron Microscopy (SEM)

Prior to imaging, the samples were freeze-dried and vertically cut in two with a sharp blade aiming at the place where the pins were. Then, these samples were Au/Pd coated by using a sputter coater (MED020, Bal-Tec, Balzers, FL, USA) with a deposition rate of 0.05 nm/s to obtain a 10 nm thicker layer. The SEM images were then recorded with a scanning electron microscope (SEM, S-4700 II, Hitachi, Tokyo, Japan).

### 2.8. Compression Testing

#### 2.8.1. Single Compression

To characterize the mechanical properties of the DN cryogels, unconfined uniaxial compression tests were performed on the samples in triplicate with an Instron^®^ 5866 electromechanical testing machine (Instron, Norwood, MA, USA), with a 50 N load cell and a head speed of 2 mm/min until the breaking points of the samples or 80% strain were reached. The samples were all cylindrical, 1 cm in diameter, and 6 mm thick.

#### 2.8.2. Fatigue Resistance

To assess the mechanical stability of the DN cryogels, cyclic stress in compression was performed on the samples in triplicate with an Instron^®^ 5866 electromechanical testing machine (Instron, Norwood, MA, USA), with a 50 N load cell and a head speed of 2 mm/min until the intended strain for each step was reached (5%, 10%, 15%, 20%, 25%, 30%, and 35%). The samples were all cylindrical, 1 cm in diameter, and 6 mm thick.

### 2.9. Cell Culture Experiments

#### 2.9.1. Cell Source and Expansion

Human chondrocytes (P2, from femoral head of an osteoarthritic patient, obtained with informed consent) were used to evaluate the cellular response to CollGTA-MeHA double network cryogels in comparison to tissue culture polystyrene (TCPS). Cells were cultured in Dulbecco’s Modified Eagles Medium—High Glucose containing 10% fetal bovine serum, 1% Penicillin-Streptomycin, and 1% non-essential amino acids. After cell seeding at a density of 2.5 × 10^4^ cells per well, in a 24-well plate, samples were incubated for 24 h at 37 °C in humidified air of 90–95% relative humidity containing 5% CO_2_.

#### 2.9.2. Evaluation of Cytotoxicity

The cytotoxicity of CollGTA-MeHA DN cryogels was investigated using a CollGTA-MeHA DN deposited in an insert in a 24-well plate after a thorough wash in PBS over several days. The cytotoxicity of the cryogels and their influence on the metabolism of chondrocytes were studied using Live/Dead staining and Cell Titer Blue assay. Live/Dead staining was performed at 24 h of culture. Samples were rinsed with cell culture medium, stained for 2 h in cell culture medium containing 3 µg/mL Calcein AM and 4 µg/mL ethidium homodimer, and rinsed with PBS. Samples were visualized using an inverted optical microscope (LSM 800, Zeiss, Germany) equipped with a 5× objective (Zeiss). The acquisition time was adjusted for each channel and red and green images were overlayed using Zen Blue Edition software. Cell Titer Blue assay was performed after 24 h. The cell medium was removed, and cells were incubated with a 10% Cell Titer Blue solution in cell culture medium for 2 h. The fluorescence signal was read using a plate reader. All fluorescence values were normalized to the fluorescence value of the cells seeded on plastic in the presence of culture medium at 24 h. All measurements were performed in triplicate.

## 3. Results and Discussion

### 3.1. Preparation of the CollGTA-MeHA Cryogels

Anisotropic collagen glutaraldehyde–hyaluronic acid methacryloyl (CollGTA-MeHA) scaffolds were fabricated by directional freezing using a pin plate set-up (Figure 1). The method consists of immobilizing copper pins in a poly (methyl methacrylate) (PMMA) plate (Supplementary Materials, Figure S1). The two materials were selected for their markedly different thermal conductivities [13,14]. The plate and the upper part of the copper pins were in contact with liquid nitrogen on one side and with the CollGTA precursor solution contained in wells on the other side. The system was insulated with Styrofoam, limiting the evaporation of the liquid nitrogen and permitting the copper pins to be the sole points initiating the freezing of the hydrogel solution. Following the freezing of the CollGTA solution, collagen crosslinking with GTA was subsequently achieved overnight at −20 °C through cryogelation, fixing the structure obtained from the pin plate. Based on preliminary tests, freezing with liquid nitrogen at −196 °C was chosen as it brought the formation of smaller pores than freezing at −20 °C. Although it has been previously suggested that the size of ice crystals decreases with increasing growth velocity, given that the suspensions were already completely frozen by the pin plate method, the subsequent freezing at a higher (less negative) temperature did not affect the organized porous structure of the cryogels [15]. After 24 h, the collagen was crosslinked around the ice crystals, and the frozen suspensions were completely thawed and removed from the mold to obtain coherent cryogel first networks. Double network cryogels were then prepared by allowing the MeHA solution to diffuse into the previously obtained anisotropic CollGTA cryogels for 48 h in the presence of a photoinitiator. The MeHA-laden CollGTA hydrogels were then photo-crosslinked under UV light and equilibrated in the PBS solution. The cryogels retained their shape without excessive swelling or shrinking (see Supplementary Materials Figure S3) after UV light exposure. A range of MeHA and CollGTA concentrations were investigated with MeHA ranging from 2 to 6% (*w*/*v*) and CollGTA ranging from 0.5 to 0.9% (*w*/*v*). All scaffolds exhibited similar structural characteristics with the pore walls protruding from the copper pins outwards, indicating that within the range of collagen and the MeHA concentration utilized the porosity orientation is driven by the growth direction of the ice crystals, whereas the components’ concentration had an effect on the mechanical properties (Supplementary Materials, Figures S6–S8). The controlled formation of the architecture of CollGTA-MeHA DN cryogels might, however, be affected by temperature, freezing time, and buffer choice [11,16,17].

### 3.2. Arcade-like Structures of CollGTA-MeHA Cryogels

Previous findings suggest that defects in cartilage architecture are a prelude to degenerative arthritis and joint wear-and-tear-related disease [18,19]. Therefore, biointerfaces in scaffolds for tissue engineering are crucial, and tuning the surface topography as well as the deep layer morphologies to be closely resembling the natural environment of chondrocytes is critical [20,21]. Moreover, chondrocytes have been previously reported to exhibit increased spreading in a micro- and nano-porous environment [22]. Consequently, engineering-controlled porous and oriented fibers could give optimal features to scaffolds for articular cartilage replacements. The architecture of collagen fibrils within articular cartilage being different in the deeper layers compared to the surface pattern is well suited to the functional requirements of the tissues and the mechanical stresses daily applied to them [23]. The particular structure of articular cartilage with an oblique organization in the middle layer, while the surface is more closely packed with smaller collagen fibrils that are oriented tangentially to the surface, only allows small molecules to diffuse, for example ions or glucose. On the contrary, in the subchondral layer the orientation is perpendicular to the joint surface. This feature has been indicated as critical to cartilage mechanical competence under complex multiaxial loading [2,19].

**Figure 2:**
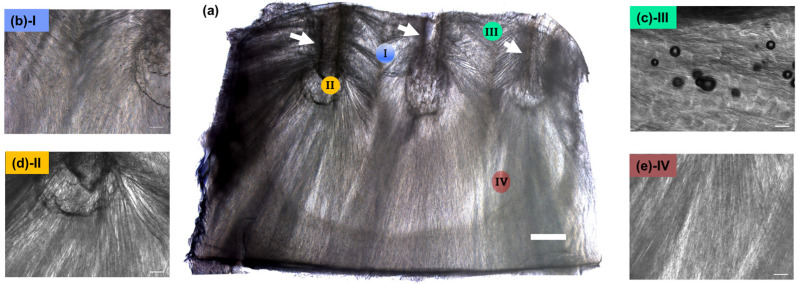
Morphological characterization of the CollGTA-MeHA cryogels by light microscopy. (**a**) Reconstructed light microscope images of the directional CollGTA-MeHA cryogels and (**b**–**e**) at higher magnification, representing (**b**) crossover areas protruding from two pins, (**c**) an area perpendicular to the pin, (**d**) where a pin was, and (**e**) an area of aligned pores. Scale bars: (**a**) 1 mm, (**b**–**e**) 200 µm.

The morphological characteristics of the obtained CollGTA-MeHA double networks were investigated by light and scanning electron microscopy. The obtained light microscopy images (Figure 2) of cross-sections of the scaffolds through the position of the pins (Figure 2a, see arrows) indicated an arcade-like structure, with an aligned arrangement protruding from the pins with an interconnectivity at the area between adjacent pins (Figure 2b). By implementing a temperature gradient along a specific direction through the pins, a controlled formation of pores and polymer fibers from the cold pins outwards was achieved. At the surface close to the pins, the collagen is more closely packed and compacted tangentially to the surface, while in the center the polymers are more elongated, obliquely oriented, and perpendicular to the surface. This arcade-like structure can be explained by the ice crystal formation occurring during the process. Ice crystals nucleated at the surface of the pin and grew outwards over a relatively large distance of more than 4 mm, which is in the range of the thickness of articular cartilages [23]. The growth rate of the ice parallel to the freezing direction was rather faster than the ice forming perpendicular to the freezing direction [24]. Consequently, this phenomenon was causing the visible lamellar structures of the crystals, thus the lamellar structure of the resulting cryogels. Moreover, since two adjacent pins form multiple cold sources, their proximity restricts the further growth of ice crystals in the direction of the neighboring pin, thus leading to the formation of the axisymmetric structure at the surface where the pins were placed.

**Figure 3:**
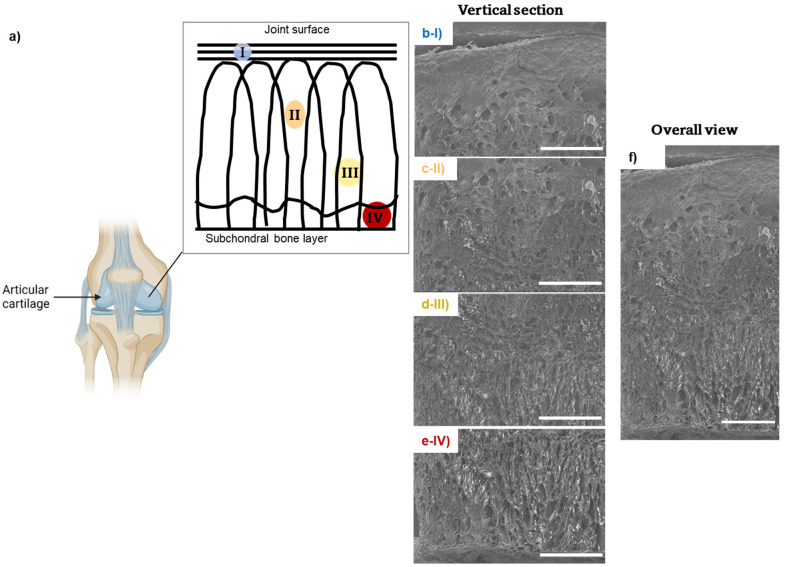
Morphological characterization of a cross-section of a DN CollGTA-MeHA cryogel by SEM, imaged from top to bottom, (**b**–**e**), respectively, following (**a**) schematic representation of the different zones of articular cartilages where (I) is the superficial zone, (II) and (III) the middle zone and (IV) the deep zone. (**f**) Stitched SEM image of (**b**–**e**). Scale bars: 1 mm.

SEM imaging of DN CollGTA-MeHA cryogels was performed to compare their morphologies to those of porcine articular cartilages after the freeze-drying process. SEM images of DN CollGTA-MeHA cryogels (Figure 3b(I)–e(IV)) revealed an architecture similar to what was previously indicated by light microscopy, namely the alignment within the constructs in an arcade-like architecture of the collagen polymers and pores. However, in addition, the freeze-dried samples revealed how these features are distributed across the sample. Because this lamellar structure was enhanced by the supplemental pore formation during the extra freeze-drying step to perform SEM imaging, the difference in the structure was confirmed. At the surface close to the pins, the collagen fibers were more closely packed and compacted tangentially to the surface, while in the center the fibers were more elongated and oriented obliquely and perpendicular to the surface with a more pronounced porous structure in the deeper zones. The pores at the surface were less visible, the middle part displayed a random organization in porosity in size and shape, and at the bottom the pores were larger and more perpendicular to the surface. The same porous distribution was visible on the SEM images of porcine articular cartilage from the literature with compact fibrils in the superficial zone and less obvious porosity, larger pores in the middle zone, random distribution of the collagen fibrils, and even larger pores in the deep zone, with aligned lamellar fibrils [25]. Therefore, the polymer fibers’ structure of the cryogels resembled those of healthy porcine articular cartilage.

Furthermore, SEM imaging seemingly showed two polymer phases, which could be tentatively attributed to CollGTA and MeHA (Figure S10). CollGTA was the first network formed and was present in lower concentration, therefore MeHA should cover the pore walls of the collagen derivative.

**Figure 4:**
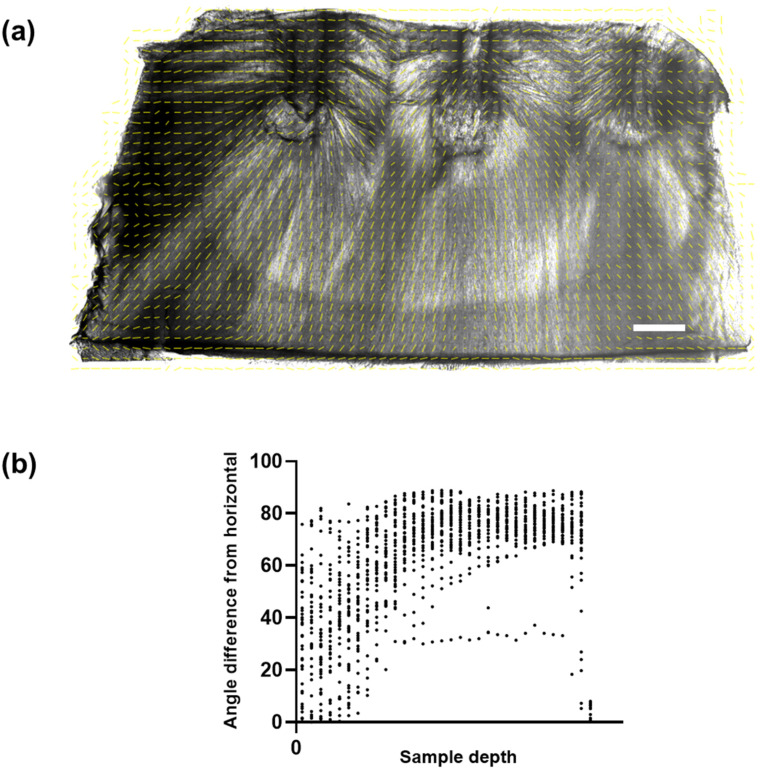
(**a**) Vector field analysis using OrientationJ plug-in of ImageJ determining local window orientation using a cubic spine algorithm where the yellow dashed lines represent the corresponding vectors. (**b**) Plot of the angular deviation from the horizontal against the sample depth on the y-axis (0 being the top of the sample). Scale bar: 1 mm.

Using the OrientationJ tool [26], the angular orientation of the fibers in the CollGTA-MeHA cryogels was quantified (Figure 4a). In the optical overlay, the analysis confirmed the presence of an arcade-like structure. To eliminate edge-effects, only the central area of the analyzed image was further processed to plot the angular orientation of the fibers against sample depth (Figure 4b). The plot clearly reflected the expected horizontal orientation of the fibers at the surface, transitioning into a vertical orientation in the lower zones. The relatively high variability in angular orientation at the surface was attributed to two main factors: firstly, the presence of holes where the pins were, and fibers extending towards and away from the field of view, creating artifacts that cannot be properly assessed by the used algorithm. Secondly, despite the large difference in thermal conductivity of the copper pins and the PMMA plate, it could be that a temperature gradient influencing the directional freezing also formed, originating at the PMMA plate holding the pins on the ceiling of the chamber. This effect results in deviations from the horizontal angle at the surface. Further iterations of the pin plate set-up could address this issue by further increasing the difference in thermal conductivity or adjusting the plate thickness.

### 3.3. Mechanical Properties of CollGTA-MeHA Cryogel Double Networks

**Figure 5:**
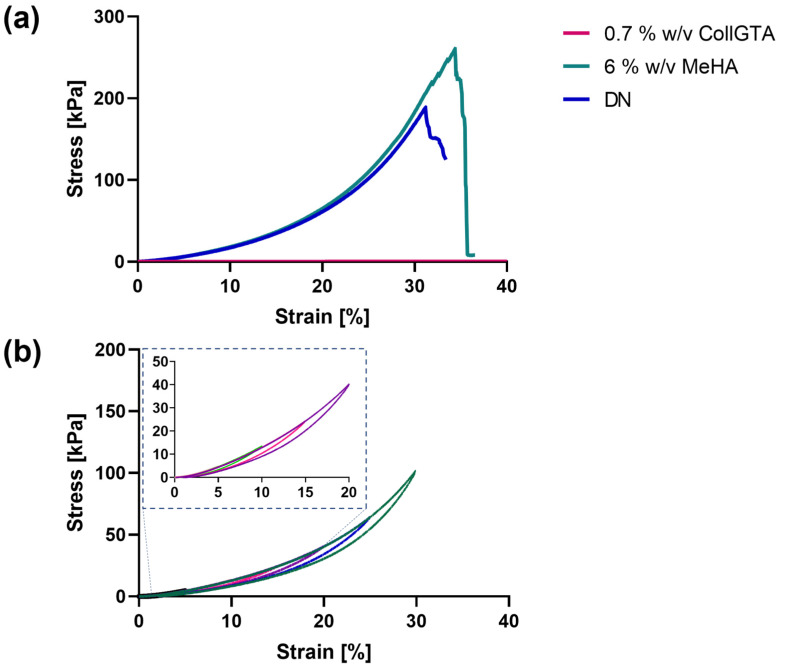
Stress–strain curves of CollGTA, MeHA single network cryogels, and CollGTA-MeHA double network cryogels. (**a**) Compressive stress–strain curves for the collagen and MeHA single networks and the corresponding CollGTA-MeHA double network with a collagen concentration of 0.7% (*w*/*v*) and MeHA concentration of 6% (*w*/*v*). (**b**) Cyclic loading of CollGTA-MeHA (0.7% (*w*/*v*) and 6% (*w*/*v*), respectively) to 30% strain in 5% increments.

Mechanical characterization was assessed by unconfined compression on the obtained CollGTA-MeHA DN cryogels and on CollGTA and MeHA single network cryogels as controls. A range of concentrations of both MeHA and CollGTA was tested to determine the optimal ratio of MeHA to CollGTA (Supplementary Materials Figures S6–S8). Figure 5a shows a graph of compressive stress versus strain for CollGTA and MeHA single networks as controls and CollGTA-MeHA DNs in unconfined compression with a strain range from 5 to 40%. The compressive stress–strain curves indicate that the CollGTA first network exhibited negligible resistance to the stress applied for the whole strain range of 5–40% and thereby exhibited a very low Young’s modulus (1.6 Pa). On the contrary, the MeHA alone exhibited a higher elastic modulus (233 kPa) and stress at break (260 kPa). Therefore, MeHA lends its good mechanical properties to the corresponding DN cryogels, which also exhibited a high early elastic modulus (~200 kPa) and a stress at break of ~184 kPa. According to previous reports, the elastic compressive modulus of articular cartilage ranges between 240 and 1000 kPa [27,28,29]. Therefore, the compressive modulus of the CollGTA-MeHA cryogels approaches the lower range of articular cartilage moduli.

We further studied the resilience of the CollGTA-MeHA cryogels by conducting multiple loading–unloading tests (Figure 5b). A small mechanical hysteresis was observed for the CollGTA-MeHA cryogels, indicating no major bond breaking during deformation as suggested by the narrow surface area in the hysteresis curves. As examples, for 5% and 30% strains, the surface areas in the curves were 1.522 kJ·m^−3^ and 172.8 kJ·m^−3^, respectively (Supplementary Materials, Table S2). Moreover, the hysteresis loading phase did not show an obvious decrease over the cycles, suggesting that energy dissipation mechanisms are reversible. The cryogels exhibited the same mechanical behavior over the different incremental strains applied, as shown by the absence of deviation in the stress–strain curves, confirming the robustness of the scaffolds.

**Figure 6:**
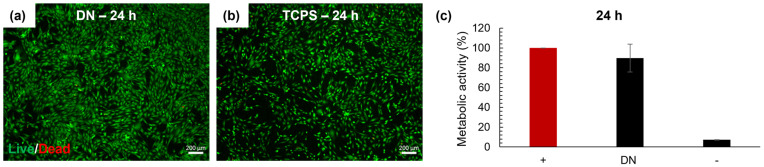
Cytotoxicity studies at 24 h. (**a**) Live/Dead microscope images of CollGTA-MeHA double network (DN), (**b**) positive control (+) TCPS, and (**c**) metabolic activity of human chondrocytes on the CollGTA-MeHA double network (DN), (+) positive control and (−) negative control. Scale: 200 µm.

Further investigation was performed to study the cryogels’ in vitro response to human chondrocytes (Figure 6). Metabolic activity of human chondrocytes after 24 h in contact with CollGTA-MeHA DN was around 90% compared to cells on TCPS as a reference. Likewise, Live/Dead staining at 24 h revealed prevalently living cells, which was in agreement with the results of the Cell Titer Blue assay. Therefore, no evident toxicity effect on human chondrocytes in contact with CollGTA-MeHA DN was found.

## 4. Conclusions

In this work, the development of DN cryogels based on collagen and hyaluronic acid derivatives displaying architectural properties similar to native articular cartilage was achieved. One limitation in the study is the use of glutaraldehyde for further cell studies. However, this can be circumvented by using less cytotoxic crosslinking agents such as genipin or EDC/NHS or a thorough wash of the constructs, as indicated by our cytotoxicity and L/D assay. Additionally, the structural and mechanical stability should be tested on a longer term, as well as the biological properties of these constructs and their capacity to support long-term cartilage tissue formation. Further development may also go towards further reinforcement of the constructs to fully match young healthy articular cartilage properties. The constructs possess enhanced mechanical properties compared to the corresponding single collagen network alone. Constructs made from 0.7% (*w*/*v*) CollGTA combined with 6% (*w*/*v*) MeHA gave a resilient cryogel. ECM-derived cryogels with organized porosity were produced from HA and collagen, although in principle the procedure could be applied to a wide range of polymers. Moreover, pore size and morphology of the scaffolds can be optimized for specific applications through established methods developed for directional freezing techniques. Overall, combining morphological, compositional, and mechanical aspects in mimicking native articular cartilage opens new perspectives concerning tissue substitutes and models with controlled architecture.

## Data Availability

Not applicable.

## Supplementary Materials

The following supporting information can be downloaded at: https://www.mdpi.com/article/10.3390/biom12121809/s1. The Supporting Information contains the synthesis details and analysis of the molecules used in this paper and complementary characterization of the cryogels (swelling ratio and shape retention study of the DN, morphological characterization, polymer diffusion in the CollGTA first network, and mechanical characterization of the cryogels). Figure S1: Schematic plan for the construction of the mould for the pin plate set-up; Figure S2: NMR spectra of methacrylate hyaluronic acid (MeHA); Figure S3: Average surface area of the cryogels after DN formation showing shape retention features; Figure S4: Light microscope images of CollGTA-MeHA double network cryogels for different concentrations of MeHA and CollGTA; Figure S5: Polymer diffusion in the first network versus time showing the optimal diffusion time needed to fully diffuse a high molecular weight polymer in a single network of CollGTA showing 48 h diffusion is needed to reach equilibrium; Figure S6: Stress-strain curves of CollGTA-MeHA double network cryogels for different concentrations and molecular weights of MeHA, keeping CollGTA concentrations at 0.7% *w*/*v*. (a–c) MeHA (MW. 280 kDa) at respectively 2, 3 and 4% *w*/*v* and (d,e) MeHA (MW. 63 kDa, referred as “vlmw”) at respectively 3 and 6% *w*/*v*; Figure S7: Stress-strain curves of CollGTA-MeHA double network cryogels for different concentrations of CollGTA, keeping MeHA concentrations at 3% *w*/*v*. (a–c) CollGTA at respectively 0.5, 0.7 and 0.9% *w*/*v* and (d–f) MeHA (MW. 63 kDa); Figure S8: Early modulus, late modulus, and stress at break of CollGTA-MeHA double network cryogels for different concentrations and molecular weights of CollGTA and MeHA, 0.7% *w*/*v* CollGTA and 6% *w*/*v* MeHA (MW. 63 kDa, referred as “vlmw”) being the best candidate; Figure S9: Characterization of the weight of MeHA diffusing in CollGTA first network. Graph of the dry weights of the first network and the second network for different concentrations of MeHA, CollGTA and different molecular weights of MeHA; Figure S10: Morphological characterization of a cross-section of the double network CollGTA-MeHA cryogels by SEM, imaged from top to bottom, b, c, d, e, respectively; Table S1. Swelling ratio of the CollGTA-MeHA double network; Table S2: Dissipation energy for different strains applied on the CollGTA-MeHA double-networks.
