# Supplementary material for: Fabrication of Collagen–Hyaluronic Acid Cryogels by Directional Freezing Mimicking Cartilage Arcade-like Structure
Source: Biomolecules. 2022 Dec 3;12(12):1809. doi: 10.3390/biom12121809 (PMC9775592; doi:10.3390/biom12121809)
Supplement: Supplementary file 1 [file biomolecules-12-01809-s001.zip › biomolecules-1994975-supplementary.pdf]

Fabrication of Collagen–Hyaluronic Acid Cryogels by Directional Freezing Mimicking Cartilage Arcade-like Structure

Taiyo Yamamoto, Rotsiniaina Randriantsilefisoa, Christoph Martin Sprecher and Matteo D'Este *

AO Research Institute Davos, Clavadelerstrasse 8, 7270 Davos, Switzerland;
* Correspondence: matteo.deste@aofoundation.org

Contents

1. Set-up 2
2. Synthesis Experiments 3
3. Results 3
2.1 Swelling ratio and shape retention of the DN 3
2.2 Morphological Characterization 5
2.3 Polymer diffusion in the CollGTA first network 7
2.4 Mechanical characterization of the cryogels 7

1. Set-up

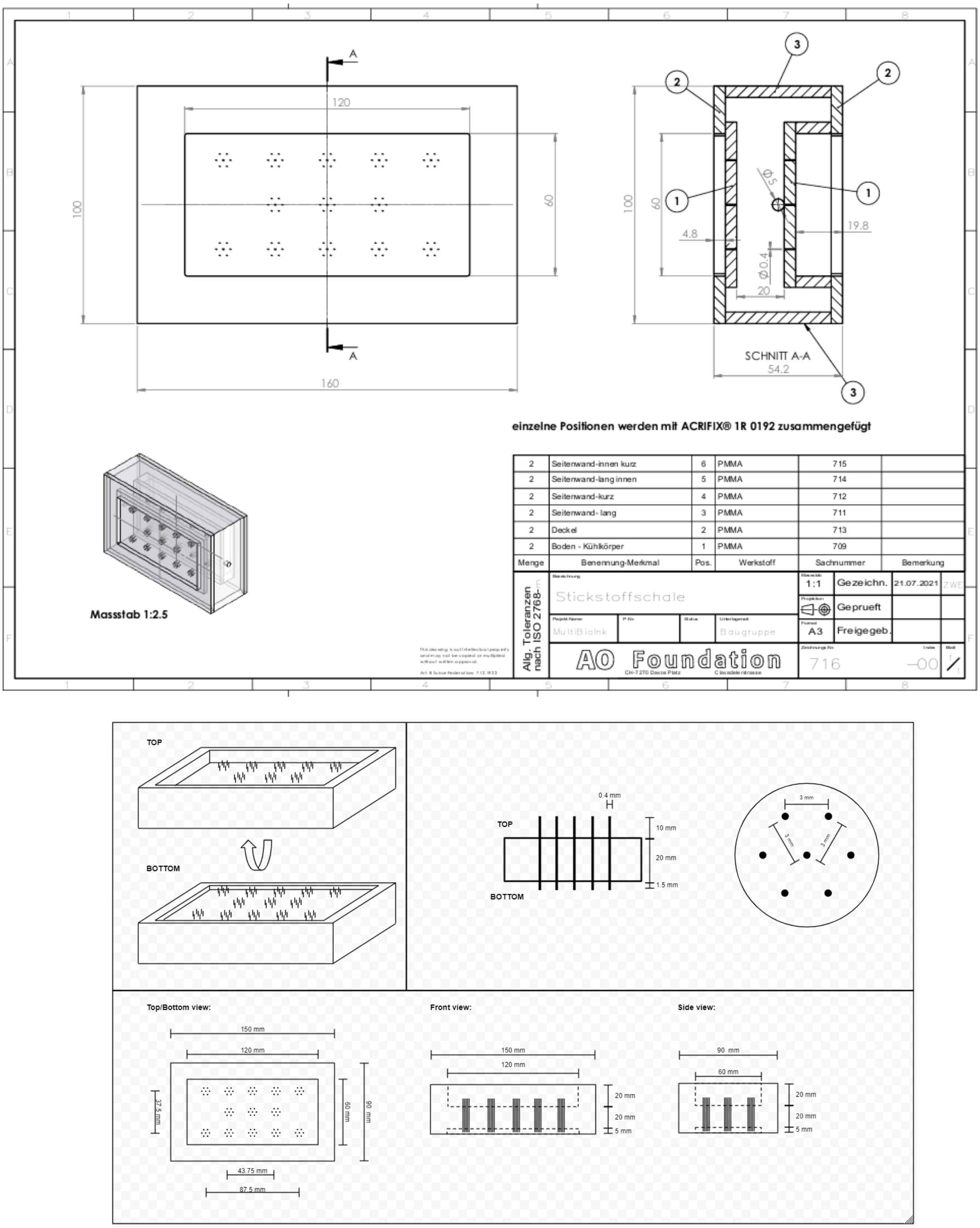

Figure S1. Schematic plan for the construction of the mould for the pin plate set-up.

2. Synthesis Experiments

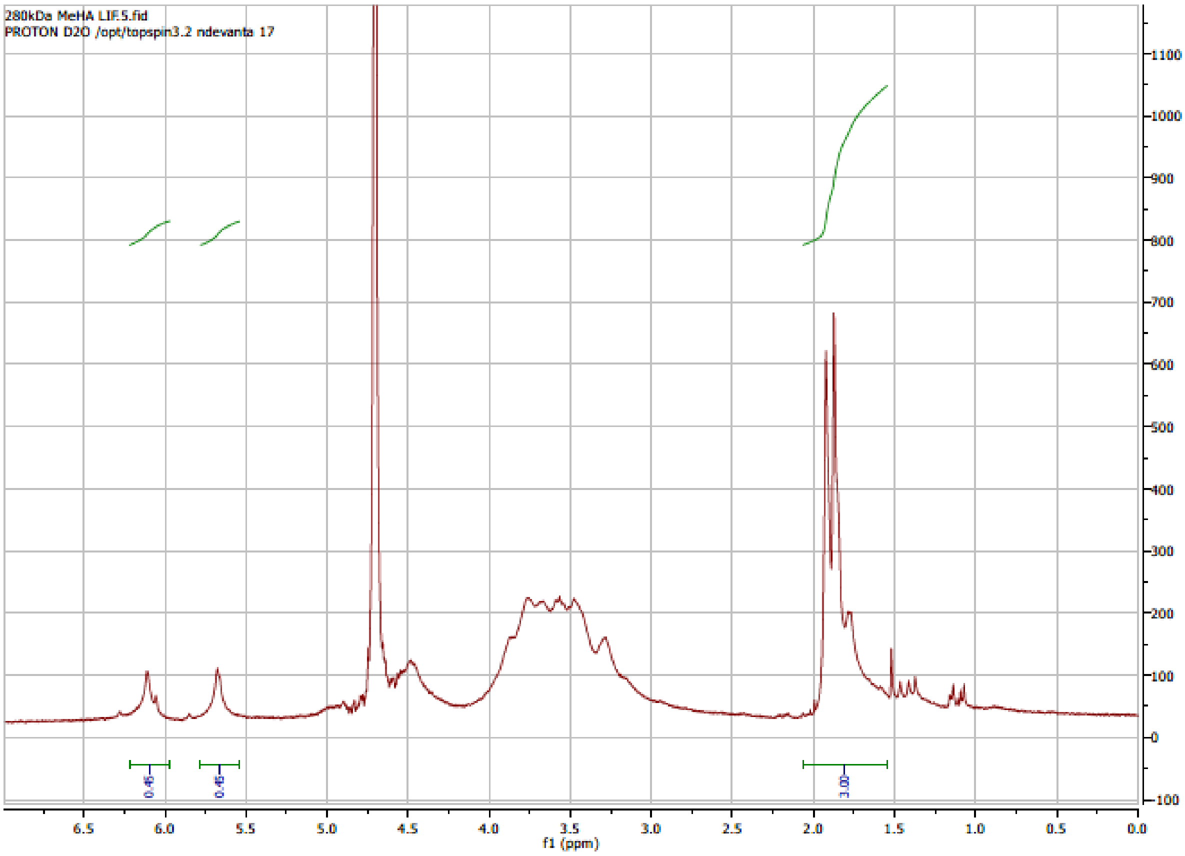

Figure S2. NMR spectra of methacrylate hyaluronic acid (MeHA).

3. Results

2.1 Swelling ratio and shape retention of the DN

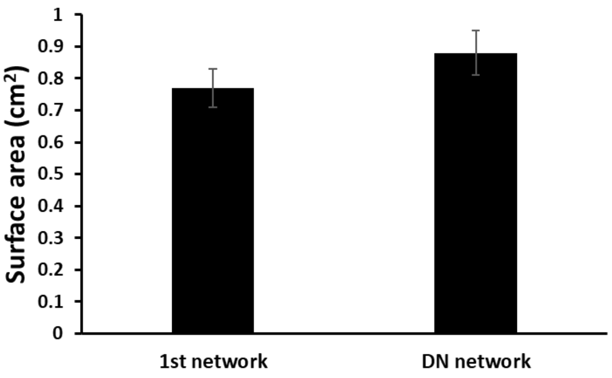

Figure S3. Average surface area of the cryogels after DN formation showing shape retention features.

Table S1. Swelling ratio of the CollGTA-MeHA double network.

	**CollGTA-MeHa**
**Swelling ratio**	16.5 +/-1.2

2.2 Morphological Characterization

0.5% CollGTA – 3% MeHA

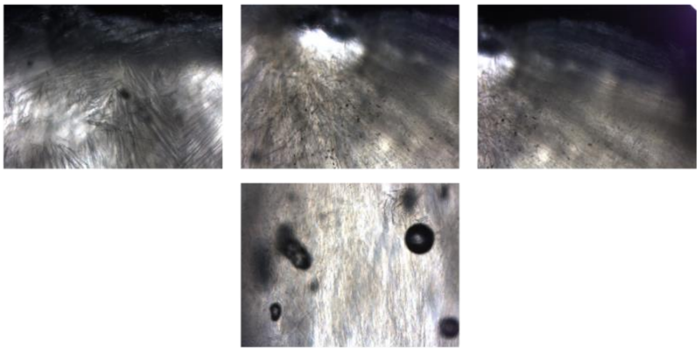

0.7% CollGTA – 2% MeHA

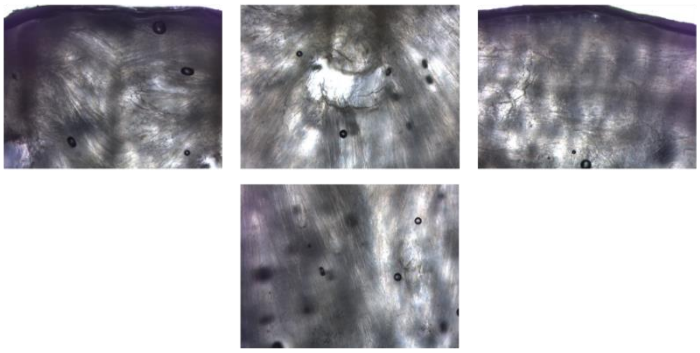

0.7% CollGTA – 4% MeHA

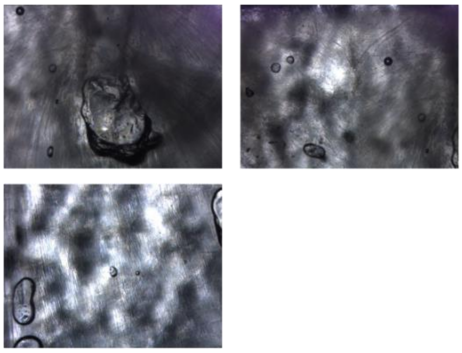

0.7% CollGTA – 6% MeHA vlmw

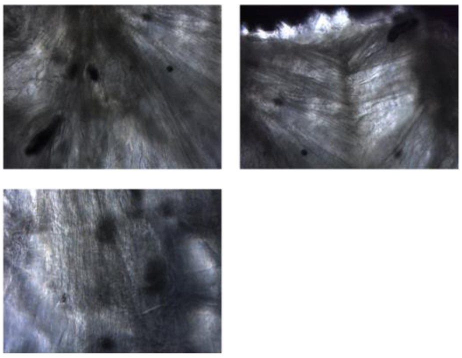

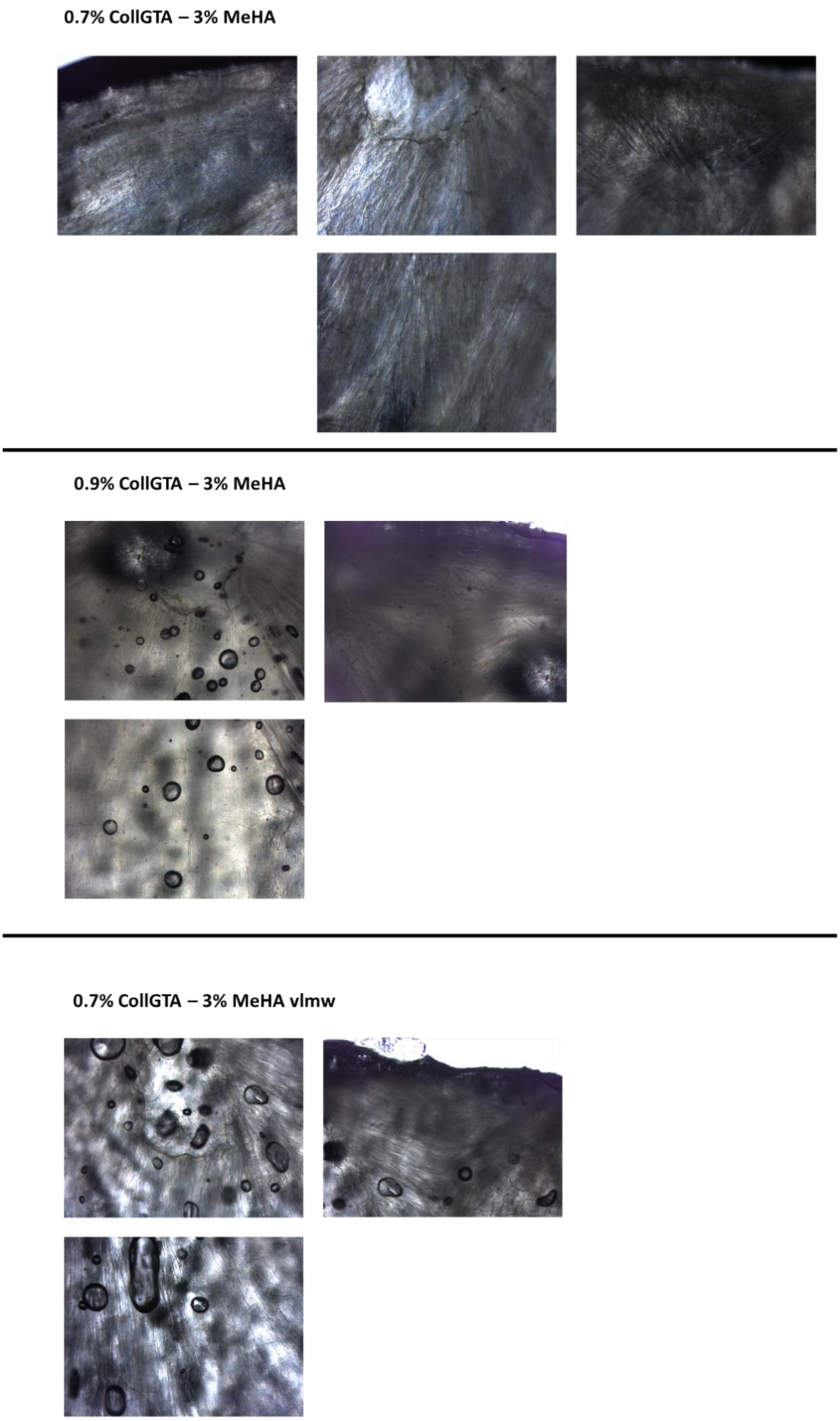

Figure S4. Light microscope images of CollGTA-MeHA double network cryogels for different concentrations of MeHA and CollGTA. Scale: 100 μm.

2.3 Polymer diffusion in the CollGTA first network

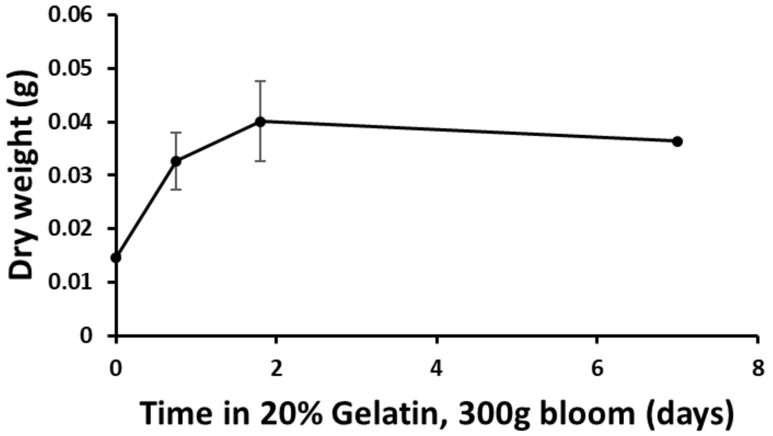

Figure S5. Polymer diffusion in the first network versus time showing the optimal diffusion time needed to fully diffuse a high molecular weight polymer in a single network of CollGTA showing 48 hours diffusion is needed to reach equilibrium.

2.4 Mechanical characterization of the cryogels

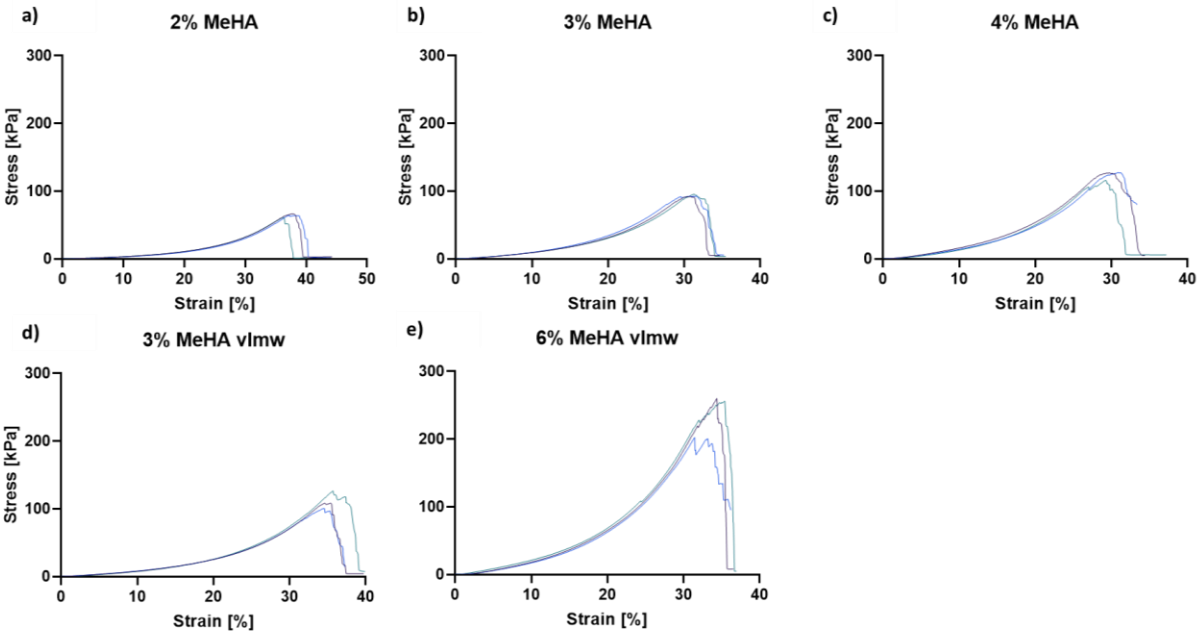

Figure S6. Stress-strain curves of CollGTA-MeHA double network cryogels for different concentrations and molecular weights of MeHA, keeping CollGTA concentrations at 0.7 %

w/v. **a)-c)** MeHA (MW. 280 kDa) at respectively 2, 3 and 4 % w/v and **d)-e)** MeHA (MW. 63 kDa, referred as "vlmw") at respectively 3 and 6 % w/v.

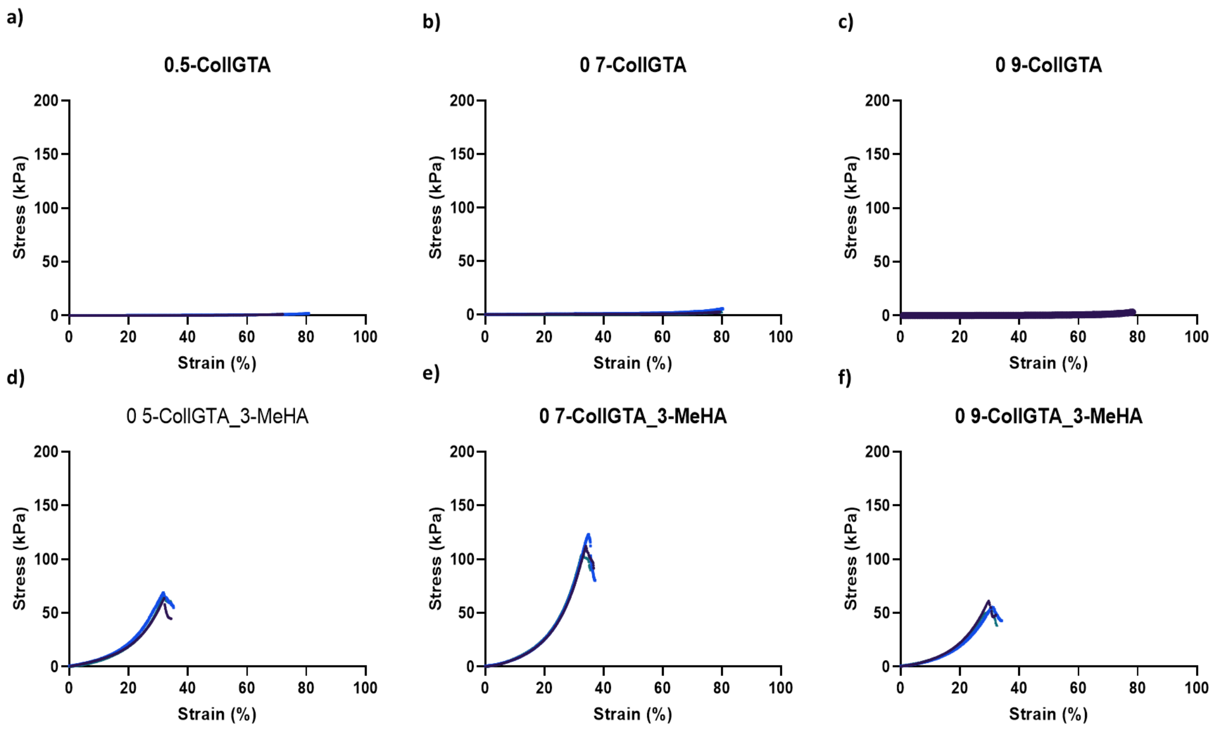

Figure S7. Stress-strain curves of CollGTA-MeHA double network cryogels for different concentrations of CollGTA, keeping MeHA concentrations at 3 % w/v. **a)-c)** CollGTA at respectively 0.5, 0.7 and 0.9 % w/v and **d)-f)** MeHA (MW. 63 kDa).

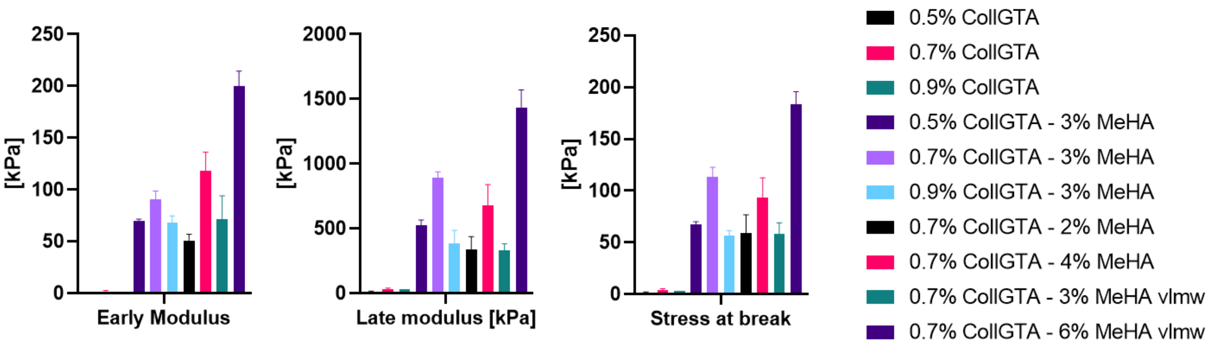

Figure S8. Early modulus, late modulus, and stress at break of CollGTA-MeHA double network cryogels for different concentrations and molecular weights of CollGTA and MeHA,

0.7 % w/v CollGTA and 6 % w/v MeHA (MW. 63 kDa, referred as "vlmw") being the best candidate.

Table S2. Dissipation energy for different strains applied on the CollGTA-MeHA double-networks.

Strain (%)	Dissipation energy (kJ.m^{-3})
5	1.522
10	8.5
15	23
20	51.4
25	98.2
30	172.8

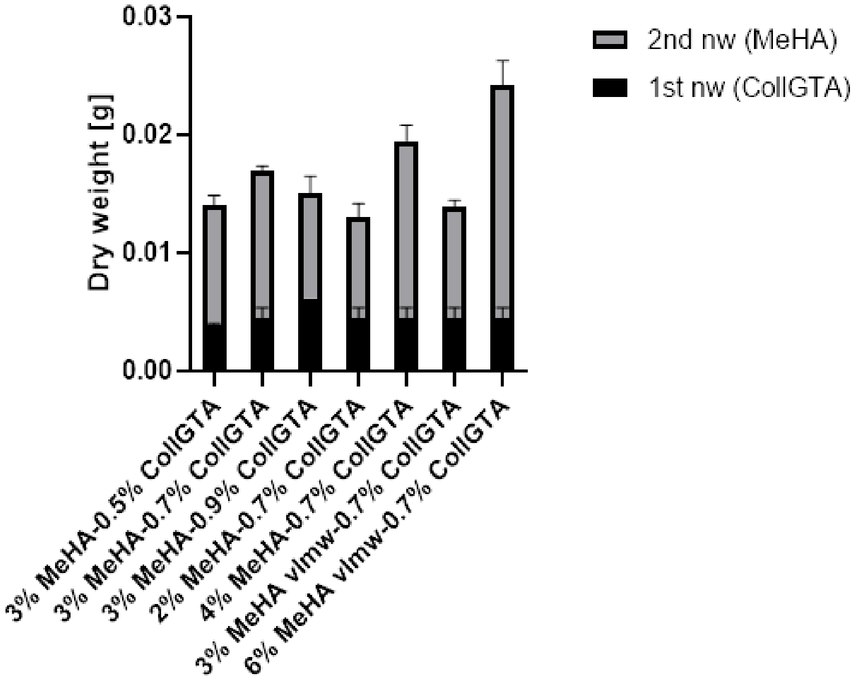

Figure S9. Characterization of the weight of MeHA diffusing in CollGTA first network. Graph of the dry weights of the first network and the second network for different concentrations of MeHA, CollGTA and different molecular weights of MeHA.

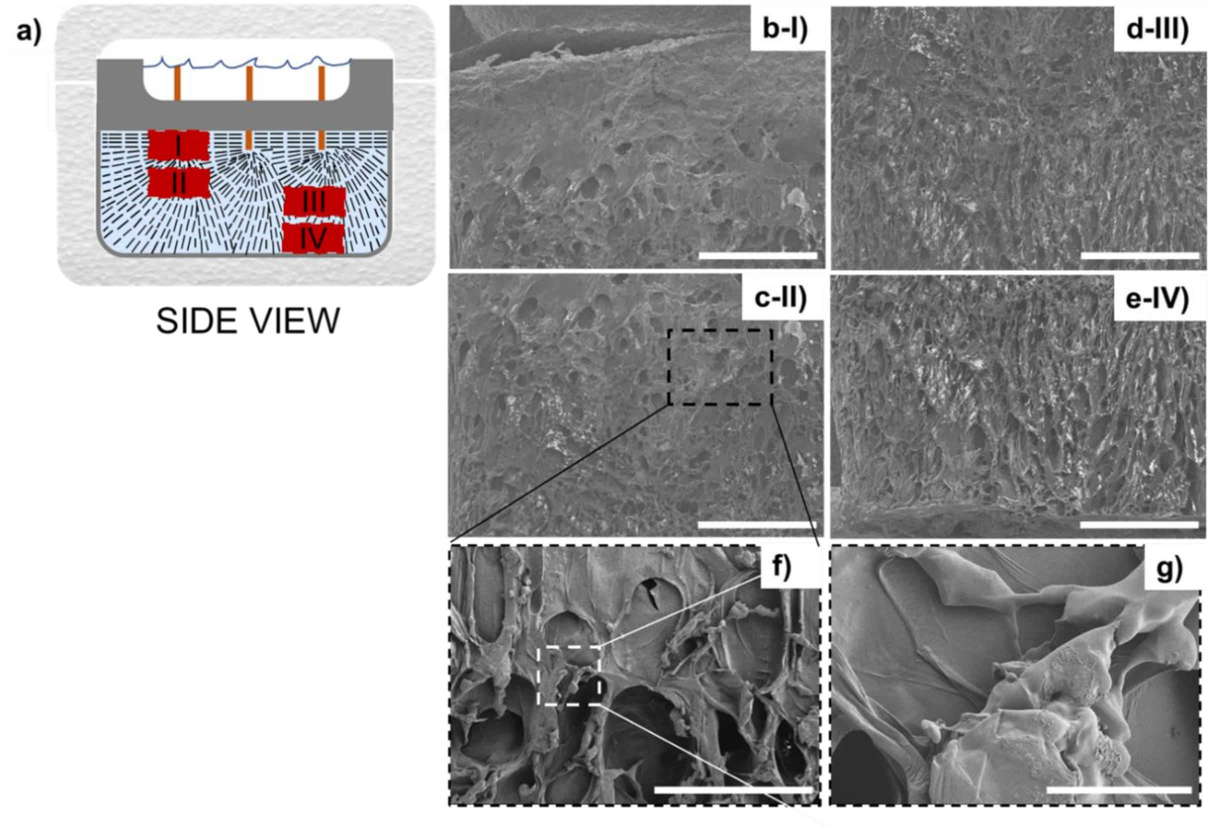

Figure S10. Morphological characterization of a cross-section of the double network CollGTA-MeHA cryogels by SEM, imaged from top to bottom, b, c, d, e, respectively. Scale bars: **b-I)-e-IV)** 1 mm, **f)** 200 µm and **g)** 50 µm.
